# Cigarettes and alcohol in relation to colorectal cancer: the Singapore Chinese Health Study

**DOI:** 10.1038/sj.bjc.6603623

**Published:** 2007-02-20

**Authors:** W H Tsong, W-P Koh, J-M Yuan, R Wang, C-L Sun, M C Yu

**Affiliations:** 1 Department of Preventive Medicine, Keck School of Medicine, University of Southern California, Los Angeles, CA 90033, USA; 2 Department of Community, Occupational and Family Medicine, Yong Loo Lin School of Medicine, National University of Singapore, Singapore 117597, Singapore; 3 The Cancer Center, University of Minnesota, Minneapolis, MN 55455, USA

**Keywords:** cigarette smoking, alcohol drinking, colorectal cancer, Singapore Chinese

## Abstract

The relations were examined between colorectal cancer and cigarette smoking and alcohol consumption within the Singapore Chinese Health Study, a population-based, prospective cohort of 63 257 middle-aged and older Chinese men and women enrolled between 1993 and 1998, from whom baseline data on cigarette smoking and alcohol consumption were collected through in-person interviews. By 31 December 2004, 845 cohort participants had developed colorectal cancer (516 colon cancer, 329 rectal cancer). Compared with nondrinkers, subjects who drank seven or more alcoholic drinks per week had a statistically significant, 72% increase in risk of colorectal cancer hazard ratio (HR)=1.72; 95% confidence interval (CI)=1.33–2.22). Cigarette smoking was associated with an increased risk of rectal cancer only. Compared with nonsmokers, HRs (95% CIs) for rectal cancer were 1.43 (1.10–1.87) for light smokers and 2.64 (1.77–3.96) for heavy smokers. Our data indicate that cigarette smoking and alcohol use interact in the Chinese population in an additive manner in affecting risk of rectal cancer, thus suggesting that these two exposures may share a common etiologic pathway in rectal carcinogenesis.

Cigarette smoking and alcohol consumption are potentially modifiable lifestyle factors that have been linked to colorectal cancer risk in prospective studies of Western populations. A meta-analysis of five cohort studies conducted in the 1980s showed a statistically significant, 30% increased risk of colorectal cancer in subjects who consumed two alcoholic drinks per day (Longnecker *et al*, 1990). More recently, a pooled analysis of eight population-based cohorts in the US, Canada, Finland, The Netherlands and Sweden, showed that daily ethanol intake of 45 g (equivalent to approximately three alcoholic drinks) or more was associated with a statistically significant 40% increase in risk of colorectal cancer overall (Cho *et al*, 2004). However, the positive alcohol-colorectal cancer association was present among smokers, but absent among nonsmokers (Cho *et al*, 2004). A possible confounding effect of smoking on the association cannot be ruled out. Recent studies have noted an up to twofold increased risk among long-term smokers (35 years or more) compared to lifelong nonsmokers, but no risk increase among shorter term smokers (reviewed by Giovannucci, 2001).

Singapore is historically a low-risk country for colorectal cancer. However, its incidence (per 100 000) has doubled among Singapore Chinese from 22.2 in 1968–72 to 42.2 in 1993–97 in males and from 16.6 in 1968–72 to 32.1 in 1993–97 in females, which are higher than comparable rates in US whites (42.2 and 29.5 per 100 000, respectively; Parkin *et al*, 2002).

There is little information, especially from prospective studies, on the possible roles of tobacco and alcohol in colorectal cancer in nonwhite populations. We investigated this subject within the Singapore Chinese Health Study, a population-based cohort study of about 60 000 Chinese men and women enrolled during 1993–1998.

## MATERIALS AND METHODS

### Study population

The design of the Singapore Chinese Health Study has been described (Hankin *et al*, 2001). Briefly, the cohort was drawn from permanent residents or citizens of Singapore who resided in government-built housing estates (86% of the Singapore population resided in such facilities during the enrollment period). The age eligibility criterion was 45–74 years. We restricted recruitment to the two major dialect groups of Chinese in Singapore, the Hokkiens and the Cantonese. Between April 1993 and December 1998, 63 257 subjects (about 85% of eligible subjects approached) were recruited. We excluded 1936 individuals with a baseline history of invasive cancer (except non-melanoma skin cancer) or superficial, papillary bladder cancer from the analysis, leaving 61 321 subjects. The study was approved by the Institutional Review Boards of the National University of Singapore, the University of Minnesota and the University of Southern California.

### Baseline exposure assessment

At recruitment, an in-person interview was conducted in the subject's home by a trained interviewer using a structured questionnaire, which covered demographics, lifetime use of tobacco (cigarettes and water-pipe), current physical activity, menstrual/reproductive history (women only), occupational exposure, medical history and family history of cancer. Information on current diet, including alcohol consumption, was assessed via a 165-item food frequency questionnaire that has been validated against a series of 24-h dietary recall interviews (Hankin *et al*, 2001) and selected biomarker studies (Seow *et al*, 1998a, 1998b) conducted on random subsets of cohort participants. The Singapore Food Composition Table, developed in conjunction with this study, allows for the computation of intake levels of roughly 100 nutritive and non-nutritive compounds per study subject (Hankin *et al*, 2001).

For each of the four types of alcoholic beverages (beer, wine, western hard liquor and Chinese hard liquor), participants were asked to choose from eight frequency categories: never or hardly, once a month, 2–3 times a month, once a week, 2–3 times a week, 4–6 times a week, once a day, and two or more times a day. Consumers were then asked to choose from four defined portion sizes. For beer, the portion sizes were one small bottle (375 ml) or less, two small bottles or one large bottle (750 ml), two large bottles, and three large bottles or more. For wine, the portion sizes were one glass (118 ml) or less, two, three and four glasses or more. For Chinese or western hard liquor, the portion sizes were one shot (30 ml) or less, two, three and four shots or more. One drink was defined as 375 ml of beer (13.6 g of ethanol), 118 ml of wine (11.7 g of ethanol), and 30 ml of western or Chinese hard liquor (10.9 g of ethanol).

In response to the following question, ‘’Have you ever smoked at least one cigarette a day for 1 year or longer', subjects who answered ‘no’ were classified as ‘nonsmokers’, those who answered ‘yes, but I quit smoking’ were classified as ‘former smokers’, and those who answered ‘yes, and I currently smoke’ were classified as ‘current smokers’. Ever smokers (former and current) were then asked about age at smoking initiation (four categories: <15, 15–19, 20–29 and 30 years or older); number of cigarettes smoked per day (six categories: 6 or less, 7–12, 13–22, 23–32, 33–42 and 43 or more); and duration of smoking (four categories; <10, 10–19, 20–39, 40 or more years).

For physical activity, subjects were asked to estimate the number of hours spent watching TV per day, and the numbers of hours per week spent on moderate activities such as brisk walking, bowling, bicycling on level ground, tai chi or chi kung, and on strenuous sports such as jogging, bicycling on hills, tennis, squash, swimming laps or aerobics.

### Case ascertainment

Incident colorectal cancer cases and deaths among cohort members was identified by linkage of the cohort database with the population-based Singapore Cancer Registry and Singapore Registry of Births and Deaths. The cancer registry has operated since 1968 and is comprehensive in coverage (Parkin *et al*, 2002). In our recent follow-up telephone/in-person interview conducted between 1999 and 2004, among the 61 685 subjects (97.5%) that we had contact with or follow-up information, either from themselves, their next-of-kin or death records, only 17 subjects (0.03%) had migrated out of Singapore. This suggests that emigration is negligible among the subjects in the cohort. As of December 31 2004 (an average of 8.9 years of follow-up), 852 cohort participants who were cancer-free at baseline had developed colorectal cancer, of which 828 (97.2%) cases were diagnosed histologically and confirmed via manual review of pathology reports by a medically trained research staff. Fourteen (1.6%) were diagnosed clinically and 10 (1.2%) cases were identified through death certificates. After excluding two lymphomas and five *in situ* carcinomas, the analysis included 845 incident cases of invasive colorectal carcinoma, of which 516 were colon and 329 were rectal/rectosigmoid cancers.

### Data analysis

For each subject, person-years of follow-up, stratified simultaneously by calendar time and age at recruitment, were counted from the date of recruitment to the date of diagnosis of colorectal cancer, death, or 31 December 2004, whichever occurred first. The person-year distribution by 5-year age groups of the entire cohort was used as an internal standard in the computation of age-adjusted incidence rate of colorectal cancer in both sexes.

Proportional hazards (Cox) regression methods were used to examine the associations between cigarette smoking/alcohol drinking and risk of colorectal cancer (Cox, 1972). Magnitude of association was assessed by the hazard ratio (HR) and its corresponding 95% confidence interval (CI) and *P* value. All Cox regression models included age at recruitment (years), gender, dialect group (Hokkien, Cantonese), year of recruitment (1993–1995, 1996–1998), level of education (no formal education, primary school, secondary or higher education), body mass index (<20–20, <24–24-<28, 28+ kg/m^2^), history of diabetes (no, yes), family history of colorectal cancer (no, yes) and weekly physical activities (no, yes). Polytomous regression methods were used to test for possible differences in exposure-risk associations by subsite (colon *vs* rectum) (Hosmer and Lemeshow, 2000). Analyses were performed for men and women separately and for both sexes combined. Because all of the studied exposure-colorectal cancer risk associations were comparable between men and women, all of the presented results were for both sexes combined with adjustment for gender.

We expressed levels of alcohol intake in units of ‘drinks’ per day to facilitate comparisons with reported data on western populations. One drink in the US is defined as one 16 oz can of beer, one glass of wine (4.5 fluid oz) or one shot (1.5 fluid oz) of hard liquor, all of which contain roughly equal amounts of ethanol (12–13 g of ethanol) (Adames, 1975). For the effect of alcohol on risk with adjustment for cigarette smoking, a three-level smoking index (never smokers, light smokers, heavy smokers) was included as a covariate in the Cox regression models. Owing to high correlation between age at starting to smoke and number of years of smoking, only one of them should be included in the multivariate models. Because age at start of smoking showed a greater effect (*P*=0.07) than duration of smoking (*P*=0.69) on goodness of fit, we chose the former to construct the level of smoking in combination with number of cigarettes per day. The ‘heavy’ smokers were those who started to smoke before 15 years of age and smoked 13 or more cigarettes per day; all remaining ever smokers were defined as light smokers. For the effect of smoking on risk with adjustment for alcohol consumption, a three-level alcohol index (nondrinkers, <7, 7+ drinks per week) was included as a covariate in the Cox regression models.

Statistical computing was conducted using SAS version 9.1 (SAS Institute Inc., Cary, NC, USA) statistical software package. All *P*-values quoted are two-sided, and *P*-values < 0.05 were considered statistically significant.

## RESULTS

Of the 61 321 cohort members free of cancer at baseline, 31% (*n*=18 738) were ever smokers, of whom 12% (*n*=2304) started to smoke before age 15 and smoked at least 13 cigarettes per day (defined as ‘heavy’ smokers). The remaining 88% (*n*=16 434) of ever smokers started to smoke at or after 15 years of age or smoked <13 cigarettes per day (defined as ‘light’ smokers). Compared with lifelong nonsmokers, ever smokers were older, were less educated, had a lower body mass index and were more likely to report a history of physician-diagnosed diabetes (Table 1).

Of the cohort participants, 19% (*n*=11 671) drank alcoholic beverages at least once a month, and among these, approximately 25% (*n*=2899) consumed seven or more drinks per week. Among them, ever smokers, on average, consumed approximately five more drinks per week than never smokers (17.3 *vs* 12.6 drinkers per week). Compared with nondrinkers, drinkers were better educated, had a lower body mass index and were less likely to report a history of physician-diagnosed diabetes (Table 1).

Among cases, the mean age at cancer diagnosis was 67.4 (s.d.=8.0) years and the mean time interval between entry into the study and cancer diagnosis was 5.5 years (range, <1 month to 11.6 years). Within the cohort, the age-adjusted incidence rate of colorectal cancer was 155 per 100 000 person-years (197 per 100 000 in men and 122 per 100 000 in women).

Table 2 shows the association between alcohol intake and risk overall and by subsite (colon, rectum) and smoking status. Compared with nondrinkers, those who consumed <7 and 7+ drinks per week had a HR of 0.96 and 1.84, respectively (*P* for trend < 0.001) after adjustment for potential confounders including cigarette smoking, physical activity and familial history of colorectal cancer. This alcohol-risk association did not differ by subsite (*P*=0.93). The associations between alcohol intake and disease risk were stronger in ever than never smokers, partly owing to the greater amount of alcoholic beverages consumed by the former. However, the interaction effect between alcohol and smoking was not statistically significant on risk of colorectal cancer overall and rectal cancer alone (both *P*>0.19), whereas the interaction effect on colon cancer risk was borderline (*P*=0.051).

Beer was the main alcoholic beverage consumed, accounting for 78% of total ethanol intake, followed by western hard liquor (16% of total ethanol intake). Consumption of wine (3% of total ethanol intake) or Chinese hard liquor (3% of total ethanol intake) was relatively rare. After adjustment for other sources of ethanol intake, the HR for colorectal cancer was 1.12 (95% CI=1.03–1.23) for one drink of beer per day and 1.25 (95% CI=0.98–1.59) for one drink of western hard liquor per day. The two HRs were not statistically different from each other (*P*=0.24).

Table 3 shows the association between cigarette smoking and overall risk and by subsite (colon, rectum). Colon cancer risk was unrelated to cigarette smoking, whereas after adjustment for potential confounders including alcohol and physical activity, rectal cancer risk was significantly associated with cigarette smoking. The risk increased with increasing amounts smoked, earlier age at starting smoking, or longer duration of smoking. The smoking-rectal cancer association was similar for former and current smokers (all *P*-values >0.63) whereas it was stronger in nondrinkers than in drinkers (Table 4). However, the interaction effect between various smoking variables and drinking status on rectal cancer risk was not significant (all *P*-values >0.67).

Table 5 shows the combined effects of smoking and alcohol consumption on risk overall and by subsite (colon, rectum). For rectal cancer, at each level of cigarette smoking, HR increased with increasing alcohol intake. Similarly, at each level of alcohol intake, risk of rectal cancer increased with increasing level of smoking. Compared with nonsmokers and nondrinkers, heavy smokers who consumed seven or more drinks of alcoholic beverages per week had a HR of 4.7 (95% CI=2.15–10.34) for rectal cancer. The interaction effects between alcohol and smoking on colorectal cancer risk (*P*=0.30), colon cancer (*P*=0.25) and rectal cancer (*P*=0.92) were not statistically significant.

Among 852 colorectal cancer cases, 24 (2.8%) cases were diagnosed clinically or identified through death certificates only. The results of the analyses after excluding these 24 cases were similar to those for the entire data set.

## DISCUSSION

Among Singapore Chinese, smoking and alcohol were independent risk factors for rectal cancer. In addition, alcohol intake also was an independent risk factor for colon cancer in this population with relatively low consumption level.

The strengths of the study are its population-based design, a large study sample size, prospective data obtained via a face-to-face interview and complete cancer case ascertainment through a comprehensive nationwide cancer registry in a small city-state with a system for easy access to specialised medical care. Cigarette smoking and alcohol consumption are highly correlated lifestyle factors across ethnically and culturally distinct populations (Koh *et al*, 2005). Therefore, residual confounding by smoking in a given alcohol-risk association and vice versa are major concerns. In the current study, the presence of an alcohol-risk association in never smokers and the presence of a tobacco-risk association in never drinkers provide important evidence that the two-lifestyle exposures exert independent effects on colorectal cancer risk.

A limitation is the lack of lifetime history of tobacco and alcohol use on study subjects. We assessed alcohol intake during the last 12 months before baseline interview and smoking histories up to the time of cohort enrollment, which may be years before the cancer diagnosis of our cases. Changes in drinking over lifetime before baseline interview and/or smoking habits between enrollment and cancer diagnosis, would result in exposure misclassification (e.g. a former drinker could be classified as a nondrinker at enrollment; a current smoker at baseline could become a former smoker at cancer diagnosis), leading to an attenuation of the estimated effects on colorectal cancer risk. Another limitation is the low alcohol consumption, especially in women, which reduces the statistical power to examine its effect on risk.

Our observed positive association between alcohol and colorectal cancer risk are consistent with the overall evidence from large, population-based Western cohorts (Kune and Vitetta 1992). Although ethanol itself is not carcinogenic, there is increasing evidence that its primary oxidative metabolite in the colon, acetaldehyde, is a potential colorectal carcinogen (Seitz *et al*, 1990; Salaspuro, 1996). Alcohol also adversely influences folate and one-carbon metabolism in the colon to contribute to abnormal DNA methylation (Giovannucci, 2004). In addition, nitrosamine precursors present in both beer and hard liquor may also play a role (Walker *et al*, 1979; Nagao *et al*, 1981). A pooled analysis of eight cohort studies in North America and Europe reported a statistically significant, 40% increase in risk of colorectal cancer among drinkers consuming three or more drinks per day. The positive alcohol-cancer risk association was comparable in men and women as well as between subsites (e.g., colon *vs* rectum; proximal *vs* distal colon) (Cho *et al*, 2004).

The Chinese populations differ in their drinking patterns from whites, but there have been few prospective studies on the effect of alcohol on colorectal cancer, whereas a meta-analysis of 14 case–control studies in China reported an overall null association (Chen *et al*, 2003). A case–control study among Singapore Chinese in the late 1980 s also failed to detect a significant association (Lee *et al*, 1989). In the Shanghai Cohort Study, a prospective cohort study of middle-aged or older men, no association between alcohol consumption and risk of death from colorectal cancer (29 cases) was found (Yuan *et al*, 1997). The present study, the first prospective investigation of alcohol use in relation to colorectal cancer in Chinese with a reasonably large number of cases and a relatively long duration of follow-up, clearly shows an independent effect of alcohol on risk, even at relatively low levels of consumption (1–2 drinks per day).

Our finding of an increased risk of rectal cancer among smokers is consistent with cohort studies in other Asian populations (Akiba and Hirayama, 1990; Shimizu *et al*, 2003) and in North America and Europe (Terry *et al*, 2001; Terry *et al*, 2002; Wei *et al*, 2004). On the other hand, our study shows a lack of association between smoking and colon cancer risk. The biological mechanism behind this subsite specificity is unknown, but differences in the embryonic tissues of the colon and rectum and in patterns of growth factors and receptors may be relevant (Chyou *et al*, 1996; Bonithon-Kopp and Benhamiche 1999; Wei *et al*, 2004). Also, nicotine may have a differential effect on colon and rectum: enhancing motility in the colon (thus reducing transit time of other carcinogens in the colon), but not in the rectum (Nyren *et al*, 1996); tobacco carcinogens may be more concentrated in the rectum than in the colon (Terry *et al*, 2002; Wei *et al*, 2004).

An additive effect of alcohol and smoking on rectal cancer risk suggests that these two exposures might share a common etiologic pathway. *N*-nitroso compounds, such as *N*-nitrosodimethylamine, *N*-nitrosodiethylamine and *N*-nitropyrolidine, occur in tobacco smoke (Tricker *et al*, 1991) and could be formed from precursors present in beer and hard liquor (Walker *et al*, 1979; Nagao *et al*, 1981). These *N-*nitroso compounds are known inducers of colorectal carcinogenesis in experimental animals (Mirvish *et al*, 2002).

In summary, smoking and alcohol were independent risk factors for rectal cancer in Singapore Chinese. The present study suggests that smoking and alcohol use interact in an additive manner on risk of rectal cancer and that alcohol rather than smoking is an independent risk factor for colon cancer in this population with relatively low exposure levels.

## Figures and Tables

**Table 1:** Distribution of selected characteristics by level of drinking and smoking, Singapore Chinese Health Study, 1993–2004

	**Cigarette smokers**			**Alcohol drinkers**		
	**Never**	**Light^a^**	**Heavy^a^**	**Nondrinker**	**<7 drinks/week**	**7+ drinks/week**
**Selected characteristics**	**(*N*=42 583)**	**(*N*=16 434)**	**(*N*=2,304)**	**(*N*=49 650)**	**(*N*=8772)**	**(*N*=2899)**
*Column percentages*						
*Gender*						
Men	27.1	83.1	91.9	37.7	69.7	85.1
Women	72.9	16.9	8.1	62.3	30.3	14.9
*Dialect*						
Cantonese	48.2	42.3	35.9	45.7	50.4	42.7
Hokkien	51.8	57.7	64.2	54.3	49.6	57.3
*Level of education*						
No formal education	29.9	20.7	22.6	29.8	15.4	18.1
Primary school	40.6	51.9	61.0	43.4	47.0	52.9
Secondary school+	29.5	27.4	16.4	26.8	37.6	28.9
*Diabetes*						
No	91.3	90.6	90.8	90.2	94.7	94.5
Yes	8.7	9.4	9.2	9.8	5.3	5.6
*Smoking status*						
Never	100	NA	NA	74.0	56.6	30.2
Former	NA	35.8	34.6	9.9	15.2	15.6
Current	NA	64.2	65.4	16.1	28.2	54.2
*Mean (±standard deviation)*						
Age (years)	55.6±7.9	58.2±8.1	58.1±7.7	56.7±8.0	54.9±7.6	55.7±7.5
Body mass index (kg/m^2^)	23.3±3.3	22.8±3.3	22.9±3.2	23.2±3.3	23.0±3.2	22.6±3.2
Age at start of smoking	NA	21.4±5.6	12.0±0.0	20.4±6.1	20.4±5.9	19.3±5.8
Cigarettes/day	NA	16.1±11.0	25.9±10.8	4.35±9.38	7.42±11.10	14.7±13.8
Drinks/week	0.43±2.30	2.10±6.38	3.40±8.84	NA	1.66±1.61	15.9±11.8

a Heavy smokers were those who started to smoke cigarettes before 15 years of age and smoked at least 13 cigarettes per day. Light smokers were those who started to smoke cigarettes at or after 15 years of age or smoked 12 or less cigarettes per day.

NA, not applicable.

**Table 2:** Alcohol consumption in relation to colorectal cancer, Singapore Chinese Health Study, 1993–2004

		**Colorectal cancer**		**Colon cancer**		**Rectal cancer**	
**Alcohol drinking**	**Person-years**	**Cases**	**HR (95% CI)^a^**	**Cases**	**HR (95% CI)^a^**	**Cases**	**HR (95% CI)^a^**
*Total subjects*							
Number of drinks/week							
Nondrinker	443968	658	1.00	416	1.00	242	1.00
<7	77374	117	0.96 (0.72–1.25)	60	0.96 (0.72–1.25)	57	1.22 (1.07–2.35)
7+	25177	70	1.84 (1.31–2.58)	40	1.84 (1.31–2.35)	30	1.59 (1.07–2.35)
* P for trend*			*0.0004*		*0.01*		*0.01*
*Never smokers*							
*Number of drinks/week*							
Nondrinker	333894	428	1.00		1.00	128	1.00
<7	44022	52	1.05 (0.78–1.44)	300	0.88 (0.60–1.30)	23	1.40 (0.89–2.22)
7+	7844	15	1.46 (0.87–2.46)	29	1.33 (0.68–2.59)	6	1.75 (0.76–4.02)
* P for trend*			*0.23*	*9*	*0.91*		*0.06*
*Ever smokers*							
Number of drinks/week							
Nondrinker	110073	230	1.00	116	1.00	114	1.00
<7	33352	65	1.06 (0.71–1.58)	31	1.06 (0.71–1.58)	34	1.11 (0.75–1.63)
7+	17333	55	1.80 (1.33–2.42)	31	2.12 (1.42–3.18)	24	1.49 (0.95–2.33)
* P for trend*			*0.0007*		*0.002*		*0.099*

a All hazard ratios (HRs) were calculated using the Cox regression models that also included the following covariates: age, gender, dialect group, year of recruitment, level of education, body mass index, history of diabetes, family history of colorectal cancer, cigarette smoking level (for total and ever smokers only), and physical exercise (see details in the Method section).

**Table 3:** Cigarette smoking in relation to risk of colorectal cancer, Singapore Chinese Health Study, 1993–2004

		**Colorectal cancer**		**Colon cancer**		**Rectal cancer**	
**Cigarette smoking**	**Person-years**	**Cases**	**HR (95% CI)^a^**	**Cases**	**HR (95% CI)^a^**	**Cases**	**HR (95% CI)^a^**
*Smoking status*							
Never smokers	385761	495	1.00	338	1.00	157	1.00
Former smokers	55933	138	1.13 (0.91–1.39)	75	0.96 (0.73–1.27)	63	1.45 (1.04–2.01)
Current smokers	104825	212	1.10 (0.92–1.32)	103	0.83 (0.64–1.06)	109	1.63 (1.23–2.17)
*Number of cigarettes/day*							
Never smokers	385761	495	1.00	338	1.00	157	1.00
<13	64149	126	1.02 (0.83–1.26)	68	0.84 (0.64–1.11)	58	1.38 (0.99–1.90)
13+	96609	224	1.18 (0.98–1.43)	110	0.91 (0.71–1.17)	114	1.71 (1.28–2.28)
*P for trend*			*0.09*		*0.38*		*0.0003*
*Age at starting to smoke*							
Never smokers	385761	495	1.00	338	1.00	157	1.00
15+ years	132319	274	1.07 (0.90–1.27)	148	0.89 (0.71–1.12)	126	1.40 (1.07–1.84)
<15 years	28439	76	1.32 (1.02–1.71)	30	0.80 (0.54–1.18)	46	2.34 (1.63–3.36)
*P for trend*			*0.06*		*0.19*		<*0.0001*
*Number of years of smoking*							
Never smokers	385761	495	1.00	338	1.00	157	1.00
<40 years	108137	177	1.05 (0.87–1.27)	94	0.88 (0.68–1.14)	83	1.37 (1.01–1.84)
40+ years	52620	173	1.19 (0.97–1.45)	84	0.87 (0.66–1.14)	89	1.85 (1.36–2.52)
*P for trend*			*0.10*		*0.27*		<*0.0001*
*Smoking level* b							
Never smokers	385761	495	1.00	338	1.00	157	1.00
Light smokers	141528	293	1.07 (0.90–1.26)	156	0.88 (0.70–1.09)	137	1.43 (1.10–1.87)
Heavy smokers	19229	57	1.49 (1.11–2.00)	22	0.89 (0.56–1.39)	35	2.64 (1.77–3.96)
*P for trend*			*0.03*		*0.28*		<*0.0001*

a All hazard ratios (HRs) were calculated using the Cox regression models that also included age, gender, dialect group, year of recruitment, level of education, body mass index, history of diabetes, family history of colorectal cancer, alcohol consumption, and physical exercise (see details in the Method section).

b Heavy smokers were those who started to smoke cigarettes before 15 years of age and smoked at least 13 cigarettes per day. Light smokers were those who started to smoke cigarettes at or after 15 years of age or smoked 12 or less cigarettes per day.

**Table 4:** Cigarette smoking in relation to risk of rectal cancer by smoking and drinking status, Singapore Chinese Health Study, 1993-2004

	**Former smokers**		**Current smokers**		**Nondrinkers**		**Drinkers**	
**Cigarette smoking**	**No. of cases**	**HR (95% CI)^a^**	**No. of cases**	**HR (95% CI)^a^**	**No. of cases**	**HR (95% CI)^a^**	**No. of cases**	**HR (95% CI)^a^**
*Smoking status*								
Never smokers	157	1.00	157	1.00	128	1.00	29	1.00
Former smokers	63	1.35 (0.96–1.85)	—	—	47	1.72 (1.18–2.52)	16	0.91 (0.48–1.72)
Current smokers	—	—	109	1.69 (1.26–2.25)	67	1.77 (1.27–2.48)	42	1.31 (0.79–2.19)
*Number of cigarettes/day*								
Never smokers	157	1.00	157	1.00	128	1.00	29	1.00
<13	16	1.01 (0.59–1.72)	42	1.58 (1.10–2.27)	42	1.56 (1.07–2.26)	16	0.97 (0.52–1.82)
13+	47	1.54 (1.06–2.24)	67	1.79 (1.28–2.50)	72	1.93 (1.37–2.73)	42	1.27 (0.76–2.12)
* P for trend*		*0.03*		*0.0004*		*0.0001*		*0.33*
*Age at starting to smoke*								
Never smokers	157	1.00	157	1.00	128	1.00	29	1.00
15+ years	46	1.16 (0.81–1.68)	80	1.55 (1.14–2.11)	87	1.63 (1.18–2.23)	39	0.97 (0.58–1.62)
<15 years	17	2.36 (1.39–4.00)	29	2.33 (1.51–3.58)	27	2.38 (1.52–3.73)	19	2.08 (1.12–3.85)
* P for trend*		*0.0082*		<*0.0001*		<*0.0001*		*0.049*
*Number of years of smoking*								
Never smokers	157	1.00	157	1.00	128	1.00	29	1.00
<40 years	39	1.15 (0.78–1.69)	44	1.58 (1.09–2.29)	58	1.66 (1.17–2.36)	25	0.88 (0.50–1.54)
40+ years	24	1.93 (1.20–3.09)	65	1.78 (1.27–2.50)	56	1.87 (1.30–2.70)	33	1.72 (0.97–3.05)
* P for trend*		*0.01*		*0.0004*		*0.0004*		*0.08*
*Smoking level* b								
Never smokers	157	1.00	157	1.00	128	1.00	29	1.00
Light Smokers	47	1.14 (0.79–1.65)	90	1.61 (1.19–2.17)	94	1.65 (1.21–2.25)	43	1.01 (0.61–1.67)
Heavy smokers	16	2.97 (1.72–5.12)	19	2.38 (1.43–3.97)	20	2.73 (1.64–4.54)	15	2.30 (1.19–4.46)
* P for trend*		*0.0036*		<*0.0001*		<*0.0001*		*0.057*

a All hazard ratios (HRs) were calculated using the Cox regression models that also included age, gender, dialect group, year of recruitment, level of education, body mass index, history of diabetes, family history of colorectal cancer, alcohol consumption (for both former and current smokers), and physical exercise (see details in the Method section).

b Heavy smokers were those who started to smoke cigarettes before 15 years of age and smoked at least 13 cigarettes per day. Light smokers were those who started to smoke cigarettes at or after 15 years of age or smoked 12 or less cigarettes per day.

**Table 5:** Joint effect of alcohol and cigarette smoking on risk of colorectal cancer, Singapore Chinese Health Study, 1993–2004

		**Smoking level**					
		**Never**		**Light^a^**		**Heavy^a^**	
**Cancer site**	**Alcohol (drinks/week)**	**Cases**	**HR (95% CI)^b^**	**Cases**	**HR (95% CI)^b^**	**Cases**	**HR (95% CI)^b^**
Colorectal cancer	Nondrinker	428	1.00	194	1.03 (0.85–1.24)	36	1.46 (1.02–2.08)
	<7	52	1.03 (0.77–1.38)	53	1.10 (0.81–1.49)	12	1.94 (1.08–3.47)
	7+	15	1.45 (0.86–2.43)	46	2.02 (1.46–2.80)	9	2.03 (1.03–3.97)
Colon cancer	Nondrinker	300	1.00	100	0.79 (0.61–1.02)	16	0.97 (0.58–1.64)
	<7	29	0.87 (0.59–1.28)	27	0.87 (0.57–1.32)	4	0.98 (0.36–2.65)
	7+	9	1.31 (0.67–2.56)	29	1.97 (1.31–2.96)	2	0.69 (0.17–2.79)
Rectal cancer	Nondrinker	128	1.00	94	1.53 (1.13–2.08)	20	2.49 (1.51–4.11)
	<7	23	1.38 (0.88–2.17)	26	1.59 (1.01–2.50)	8	3.90 (1.87–8.15)
	7+	6	1.76 (0.77–4.02)	17	2.20 (1.29–3.76)	7	4.71 (2.15–10.34)

a Heavy smokers were those who started to smoke cigarettes before 15 years of age and smoked at least 13 cigarettes per day. Light smokers were those who started to smoke cigarettes at or after 15 years of age or smoked 12 or less cigarettes per day.

b All hazard ratios (HRs) were calculated using the Cox regression models that also included age, gender, dialect group, year of recruitment, level of education, body mass index, history of diabetes, family history of colorectal cancer, and physical exercise (see details in the Method section).
